# Leisure-time physical activity as a key protective factor against cognitive decline in older adults: an isotemporal substitution analysis

**DOI:** 10.1590/0102-311XEN046523

**Published:** 2024-07-29

**Authors:** Flávio Rodrigues Duarte, Lucas Lima Galvão, Rizia Rocha-Silva, Sheilla Tribess, Rafaela Gomes dos Santos, Douglas de Assis Teles Santos, Jair Sindra Virtuoso

**Affiliations:** 1 Universidade Federal do Triângulo Mineiro, Uberaba, Brasil.; 2 Universidade do Estado da Bahia, Teixeira de Freitas, Brasil.

**Keywords:** Exercise, Sedentary Lifestyle, Cognitive Dysfunction, Exercício Físico, Comportamento Sedentário, Disfunção Cognitiva, Ejercicio Físico, Conducta Sedentaria, Disfunción Cognitiva

## Abstract

This study aimed to test hypothesized effects of replacing sedentary behavior with moderate-to-vigorous physical activity, sleep, and different domains of physical activity by equivalent amounts on suggestive cognitive decline in an older adult population. This was a cross-sectional study including 473 older adults aged ≥ 60 years. Cognitive decline was assessed using the *Mini-Mental Health Examination*. Physical activity, its different domains and the time of exposure to sedentary behavior were assessed using the *International Physical Activity Questionnaire*. For data analysis, two isotemporal substitution models were constructed using Poisson regression. The first model tested the effect of sleep time, sedentary behavior, and moderate-to-vigorous physical activity on cognitive decline. The second model was used to determine the effect of physical activity domains (leisure, work, transport, and home), sleep time, and sedentary behavior on cognitive decline. Physical activity during leisure time was protective against cognitive decline among all domains tested, replacing sedentary behavior, sleep, and transport. Conversely, substitution of the leisure domain for sedentary behavior, sleep, and transport was considered a risk factor for cognitive decline. Leisure time proved to be a strong protective factor in reducing the risk of cognitive decline, and it is necessary to encourage and stimulate public policies that include it.

## Resumo

Este estudo objetivou verificar os efeitos hipotéticos da substituição do comportamento sedentário por atividade física moderada a vigorosa, sono e diferentes domínios da atividade física por quantidades equivalentes sobre o declínio cognitivo em uma população idosa. Trata-se de um estudo transversal, com 473 idosos de ≥ 60 anos. O declínio cognitivo foi avaliado por meio do *Mini Exame do Estado Mental*. A atividade física, seus diferentes domínios e o tempo de exposição ao comportamento sedentário foram avaliados por meio do *Questionário Internacional de Atividade Física*. Para a análise dos dados, foram utilizados dois modelos de substituição isotemporal, por meio da regressão de Poisson. O primeiro modelo testou o efeito do tempo de sono, comportamento sedentário e atividade física moderada a vigorosa no declínio cognitivo. O segundo modelo foi utilizado para determinar o efeito dos domínios de atividade física (lazer, trabalho, transporte e casa), tempo de sono e comportamento sedentário no declínio cognitivo. A atividade física no lazer foi um fator protetivo contra declínio cognitivo em todos os domínios testados, substituindo comportamento sedentário, sono e transporte. Por outro lado, a substituição do domínio lazer por comportamento sedentário, sono e transporte foi considerada fator de risco para o declínio cognitivo. O tempo livre mostrou-se um forte fator protetor na redução do risco de declínio cognitivo, sendo necessário incentivar e estimular políticas públicas.

## Resumen

Este estudio tuvo como objetivo verificar los efectos hipotéticos al reemplazar el comportamiento sedentario por actividad física de moderada a vigorosa, sueño y diferentes dominios de actividad física por cantidades equivalentes sobre el deterioro cognitivo en una población anciana. Se trata de un estudio transversal, con 473 ancianos ≥ 60 años. El deterioro cognitivo se evaluó mediante el *Mini Examen del Estado Mental*. La actividad física, sus diferentes dominios y el tiempo de exposición al comportamiento sedentario se evaluaron mediante el *Cuestionario Internacional de Actividad Física*. Para el análisis de los datos, se utilizaron dos modelos de reemplazo isotemporal, utilizando la regresión de Poisson. El primer modelo probó el efecto del tiempo de sueño, comportamiento sedentario y actividad física de moderada a vigorosa en el deterioro cognitivo. El segundo modelo se utilizó para determinar el efecto de los dominios de actividad física (ocio, trabajo, transporte y hogar), tiempo de sueño y comportamiento sedentario en el deterioro cognitivo. La actividad física en el tiempo libre fue un factor protector contra el deterioro cognitivo en todos los dominios evaluados, reemplazando el comportamiento sedentario, el sueño y el transporte. Por otra parte, reemplazar el dominio del ocio por comportamiento sedentario, sueño y transporte se consideró un factor de riesgo para el deterioro cognitivo. El tiempo libre demostró ser un fuerte factor protector para reducir el riesgo de deterioro cognitivo, por lo que es necesario fomentar y estimular políticas públicas.

## Introduction

The aging process is associated with a gradual decline in cognitive function, and older adults commonly notice that tasks requiring memory take longer to complete ^1^. Cognitive decline is a complex process. Therefore, reducing or eliminating risk factors and enhancing protective factors are potential interventions to minimize losses during aging, contributing to greater independence, improved memory, and reduced risk of dementia ^2^.

Scientific evidence shows that age, sex, disease, social and economic factors, poor nutrition, low levels of physical activity, and prolonged sedentary behavior are factors associated with cognitive decline ^3^ ^,^ ^4^. Regular physical activity promotes improvements in physical, psychological, and metabolic functioning and helps reverse the effects of chronic diseases ^5^ ^,^ ^6^. However, with technological advances, physical activity levels have decreased significantly, and sedentary behavior has become a public health problem ^7^.

Millions of deaths are recorded every year as a result of physical inactivity ^8^. Global and Brazilian recommendations for physical activity have been reported previously ^9^ ^,^ ^10^. Studies have shown that physical activity improves physical and cognitive health in older adults ^11^ ^,^ ^12^, with a systematic review demonstrating that aerobic, strengthening, and walking exercises can provide cognitive benefits ^13^. The challenge is to increase the participation of older adults in regular physical activity, as few follow the recommended guidelines ^14^.

Despite the established association between sedentary time and cognitive decline in older adults, the specific causal relationship needs further investigation. Notably, evidence from a systematic review indicates a negative association between sedentary behavior and cognitive decline, suggesting that limiting sedentary time and engaging in moderate-to-vigorous physical activity may promote healthier cognitive aging ^15^.

Studies have suggested that reducing daily sedentary time and doing moderate-to-vigorous physical activity, light physical activity, or sleeping may provide important benefits to the physical and cognitive health of older adults ^16^ ^,^ ^17^. However, we are not aware of any studies that have examined the effect of the substitution of sedentary behavior by physical activity domains proposed by the *International Physical Activity Questionnaire* (IPAQ) on cognitive decline in the older adult population.

Therefore, this study aimed to test the hypothetical effects of replacing sedentary behavior with moderate-to-vigorous physical activity, sleep, and different physical activity domains by equivalent amounts on cognitive decline in an older adult population. We hypothesized that replacing sedentary behavior with moderate-to-vigorous physical activity would reduce the risk of cognitive decline and that different physical activity domains would lower the risk of cognitive decline by reducing sleep time and sedentary behavior.

## Methods

### Study design

This is an analytical, cross-sectional, observational study using exploratory research methods based on household surveys. The data for this study were extracted from the baseline (2015) of the *Longitudinal Study of Elder Health in Alcobaça* (ELSIA). This study aimed to monitor the older adults evaluated in this phase and to establish the relationship between the behavioral aspects and health status of older adults living in the municipality of Alcobaça, Bahia State, Brazil.

### Participants

Older adults were contacted during home visits, using data provided by the Municipal Health Department of Alcobaça. They were informed of the objectives and requested to participate voluntarily in the research. Upon acceptance, an informed consent form was given and a questionnaire was administered in the form of an interview by duly trained academics and physical education professionals. Data collection took place from July to September 2015. The interview script was previously tested in a pilot study (to identify psychometric indices) and constructed throughout the composition of other instruments.

A total of 743 older adults registered in the Family Health Strategy of the Brazilian Ministry of Health were contacted for inclusion in the study. During data collection, 54 older adults refused to participate in the study, 58 older adults were excluded for not meeting the inclusion criteria, and 158 older adults were excluded after three contact attempts. Therefore, in total, 473 adults of both sexes aged ≥ 60 years participated in this study.

To calculate sample representativeness, the criteria of Luiz & Magnanini ^18^ for finite populations were applied, considering a 50% estimated prevalence of mild suggestive cognitive decline, a 4% tolerable sampling error, an 20% increment for adjusted analysis, and a 10% addition to compensate for losses.

Exclusion criteria were as follows: severe cognitive impairment (≤ 11 points) according to the *Mini-Mental State Examination* (MMSE) ^19^ adapted for the Brazilian population ^20^; severe visual and hearing impairment; wheelchair use; severe sequelae of stroke with localized loss of strength; and presence of a terminal illness.

**Figure 1:**
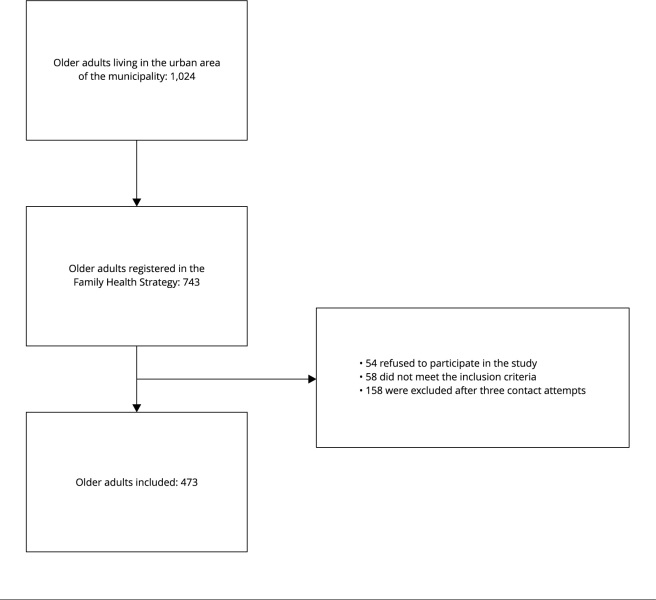
Study flowchart.

A comprehensive description of the data collection procedures can be found in other studies ^21^ ^,^ ^22^ and in the Figure 1.

### Suggestive cognitive decline

Cognitive decline was assessed during an interview using the MMSE. This instrument assesses the status of cognitive functions and is useful in screening for cognitive alterations ^19^ ^,^ ^20^. In addition, older adults were divided into two groups - those with and without cognitive decline ^23^ - using a cut-off point of 18/19 for illiterate individuals and 24/25 for literate individuals.

### Physical activity and sedentary behavior

Physical activity was assessed using the long version of the IPAQ ^24^, validated for the Brazilian older adult population ^25^ ^,^ ^26^. Physical activity level was determined based on moderate-to-vigorous physical activity performed for at least 10 consecutive minutes and assessed in the physical activity domains of leisure, work, transport, and home. For characterization, the population was dichotomized into sufficiently and insufficiently active ^9^.

Sedentary behavior was assessed using questions about time spent in sitting, lying, and reclining positions. The total time spent sitting (minutes/day) was calculated as the weighted arithmetic mean of the time spent sitting on a weekday and on a weekend day: [(time spent sitting on a weekday × 5 + time spent sitting on a weekend day × 2) / 7]. Sitting time was considered high from the 75th percentile (540 minutes/day) ^21^.

### Sleep assessment

Night sleep duration was measured using a specific question from the *Pittsburgh Sleep Quality Index* ^27^ validated for Brazilians (PSQI-BR) ^28^: “During the past month, how many hours of actual sleep did you get at night? (This may be different from the number of hours you spent in bed.)”. This question was used to calculate the total time of activities during the day.

### Covariates

The covariates evaluated were sex (male and female), age range (60-69 years, 70-79 years, or ≥ 80 years), marital status (without a partner or with a partner), continuous medication use (yes or no), alcohol consumption (yes or no), hospitalization in the previous 12 months (yes or no), family arrangement (alone or accompanied), and depressive symptoms (absent or present).

Frailty was identified according to the criteria proposed by the *Study of Osteoporotic Fractures* (SOF). The following question was used to assess unintentional weight loss: “In the past year, have you lost more than 4.5kg unintentionally (i.e., no diet or exercise)?”. Self-reported fatigue was assessed using the following two questions from the *Geriatric Depression Scale* - GDS (short form) adapted for the Brazilian population: “Have you stopped doing many of your activities and interests?” and “Do you feel full of energy?”; a positive response to the first question or a negative response to the second question were considered signs of exhaustion or fatigue. One last factor was then added to the assessment of frailty: loss of strength, defined by the inability to stand up from a chair five times without using the arms, according to a test performed. Older adults who presented with 2 or 3 of these components (unintentional weight loss, self-reported fatigue, and loss of strength) were classified as frail, and the others were classified as non-frail ^29^.

Depressive symptoms were assessed using the reduced version of the GDS-15 ^30^, translated and validated for the Brazilian population ^31^. This scale consists of 15 positive/negative questions about satisfaction with life, happiness, and social interaction. The total score for the GDS-15 ranges from 0 to 15 points. The higher the score, the greater the severity of depressive symptoms. The cut-off point for the presence of depressive symptoms was six points or more (≥ 5 points).

### Data analysis

Epidata software version 3.1b (http://www.epidata.dk/) was used to create the database. Analyses were carried out using SPSS 23.0 program (https://www.ibm.com/). Descriptive statistics, absolute and relative frequencies, dispersion calculations, and chi-squared inferential statistics were used to characterize the study subjects in order to analyze the associations between covariates and cognitive decline.

Two isotemporal substitution models were constructed ^32^ ^,^ ^33^. The first model tested the effect of sleep time, sedentary behavior, and moderate-to-vigorous physical activity on cognitive decline. The second model was used to determine the effect of physical activity domains (leisure, work, transport, and home), sleep time, and sedentary behavior on cognitive decline.

Isotemporal substitution analyses were performed using Poisson regression with an estimate of the prevalence ratio (PR) and 95% confidence interval (95%CI). In this analysis, the time spent on the observed activities was divided by the constant (time unit) used to test the effect. A new variable was created with the sum of the time spent on all activities. Then, the activity intended to show the effect of being replaced was removed from the model. All the others, including the total discretionary time constant, remained in the model ^32^, as shown in the following equation:

Cognitivedecline=b1physicalactiviy∈work+b2physicalactiviy∈home+b3physicalactiviy∈leisure+b4physicalactiviy∈transport+b5sleeptime+b6time∈sedentarybehavior+b7totaltime+b8covariates

The effects of substitutions at 10, 20, 30, 40, 50, and 60 minutes in the presence of cognitive decline were tested. The models were adjusted for sex, age range, hospitalization, presence of depressive symptoms, and frailty. A 5% (p ≤ 0.05) significance level was used.

### Ethical procedures

The study protocol and procedures were conducted in accordance with the *Declaration of Helsinki*. They were approved by the Ethics Committee in Human Research of the Federal University of Triângulo Mineiro (n. 966,983/2015, CAAE: 41401015.0.0000.5154). Older adults who agreed to participate in the study signed an informed consent form.

## Results

Among the participants with cognitive decline, most were female (n = 206), aged ≥ 80 years (n = 54), did not drink alcohol (n = 165), and did not have a partner (n = 168). Table 1 shows the characteristics of the sample based on suggestive cognitive decline.

**Table 1:** Sociodemographic, health, and behavioral characteristics according to suggestive cognitive decline.

Characteristics	Total	Suggestive cognitive decline		Valor de p
		Normal	Suggestive decline	
	n (%)	n (%)	n (%)	
Sex				< 0.001
Male	177 (37.4)	101 (57.1)	76 (42.9)	
Female	296 (62.6)	90 (30.4)	206 (69.6)	
Age range (years)				0.015
60-69	261 (55.2)	111 (42.5)	150 (57.5)	
70-79	140 (29.6)	62 (44.3)	78 (55.7)	
≥ 80	72 (15.2)	18 (25.0)	54 (75.0)	
Marital status				0.003
Without a partner	255 (54.0)	87 (34.1)	168 (65.9)	
With a partner	217 (46.0)	103 (47.5)	114 (52.5)	
Family arrangement				0.937
Alone	76 (16.1)	31 (40.8)	45 (59.2)	
Accompanied	397 (83.9)	160 (40.3)	237 (59.7)	
Hospitalization				0.185
No	393 (83.1)	164 (41.7)	229 (58.3)	
Yes	80 (16.9)	27 (33.8)	53 (66.3)	
Depressive symptoms				0.181
Absent	417 (88.2)	173 (41.5)	24 (58.5)	
Present	56 (11.8)	18 (32.1)	38 (67.9)	
Medication use				0.996
No	99 (20.9)	40 (40.4)	59 (59.6)	
Yes	374 (79.1)	151 (40.9)	223 (59.6)	
Alcohol consumption				0.001
No	247 (52.2)	82 (33.3)	165 (66.8)	
Yes	226 (47.8)	109 (48.2)	117 (51.8)	
Level of physical activity (minutes/week)				0.113
≥ 150	249 (52.6)	109 (43.8)	140 (56.2)	
< 150	224 (47.4)	82 (36.6)	142 (63.4)	
Sedentary behavior (minutes/day)				0.128
< 540	354 (74.8)	150 (42.4)	204 (57.6)	
≥ 540	119 (25.2)	41 (34.5)	78 (65.5)	
Frailty				0.365
Non-frail	417 (91.4)	170 (40.8)	247 (59.2)	
Frail	39 (8.6)	13 (33.3)	26 (66.7)	

Table 2 shows the isotemporal substitution models for sleep time, sedentary behavior, and moderate-to-vigorous physical activity. At all time points analyzed, no associations with cognitive decline were observed among the proposed substitutions (p > 0.005).

**Table 2:** Isotemporal substitution model of the association of sleep time reallocation, sedentary behavior, and moderate to vigorous physical activity with cognitive suggestive decline.

Substitution template	Suggestive decline		
	PR (95%CI)	PR (95%CI)	PR (95%CI)
	Moderate-to-vigorous physical activity	Sleep	Sedentary behavior
10 minutes			
Substitution of moderate-to-vigorous physical activity	-	1.01 (0.99-1.02)	1.00 (0.99-1.01)
Substitution of sleep	0.99 (0.97-1.00)	-	0.99 (0.98-1.00)
Substitution of sedentary behavior	0.99 (0.98-1.01)	1.00 (0.99-1.01)	-
20 minutes			
Substitution of moderate-to-vigorous physical activity	-	1.02 (0.99-1.05)	1.00 (0.97-1.02)
Substitution of sleep	0.97 (0.95-1.00)	-	0.98 (0.97-1.00)
Substitution of sedentary behavior	0.99 (0.96-1.02)	1.01 (0.99-1.02)	-
30 minutes			
Substitution of moderate-to-vigorous physical activity	-	1.03 (0.98-1.07)	1.00 (0.96-1.04)
Substitution of sleep	0.96 (0.92-1.01)	-	0.97 (0.95-1.00)
Substitution of sedentary behavior	0.99 (0.95-1.02)	1.02 (0.99-1.04)	-
40 minutes			
Substitution of moderate-to-vigorous physical activity	-	1.04 (0.98-1.10)	1.01 (0.96-1.06)
Substitution of sleep	0.95 (0.90-1.01)	-	0.97 (0.94-1.00)
Substitution of sedentary behavior	0.98 (0.93-1.03)	1.02 (0.99-1.06)	-
50 minutes			
Substitution of moderate-to-vigorous physical activity	-	1.05 (0.98-1.13)	1.01 (0.95-1.08)
Substitution of sleep	0.94 (0.88-1.01)	-	0.96 (0.93-1.00)
Substitution of sedentary behavior	0.98 (0.92-1.05)	1.03 (0.99-1.07)	-
60 minutes			
Substitution of moderate-to-vigorous physical activity	-	1.06 (0.97-1.15)	1.02 (0.92-1.08)
Substitution of sleep	0.93 (0.86-1.02)	-	0.95 (0.91-1.00)
Substitution of sedentary behavior	0.97 (0.90-1.06)	1.04 (0.99-1.09)	-

95%CI: 95% confidence interval; PR: prevalence ratio.

Note: adjusted for sex, hospitalization, family arrangement, depressive symptoms, and frailty.

Table 3 shows the isotemporal substitution model arranged by physical activity, sedentary behavior, and sleep domains. No associations were observed between work and home domains (p > 0.005). Physical activity during leisure time proved to be protective among all domains tested, substituting sedentary behavior, sleep, and transport time (p < 0.005). Conversely, the substitution of leisure time for sedentary behavior, sleep, and transport time was found to be a risk factor for cognitive decline (p < 0.005).

**Table 3:** Isotemporal substitution model of the association of sleep time reallocation, sitting, and physical activity domains with suggestive cognitive decline.

Substitution templates	PR (95%CI)	PR (95%CI)	PR (95%CI)	PR (95%CI)	PR (95%CI)	PR (95%CI)
	Work	Home	Leisure	Transportation	Sleep	Sedentary behavior
10 minutes						
Substitution of work	-	0.98 (0.95 (1.02)	0.94 (0.89-1.00)	1.02 (0.97-1.08)	1.00 (0.98-1.03)	1.00 (0.97-1.02)
Substitution of the home domain	1.01 (0.97-1.04)	-	0.95 (0.90-1.01)	1.03 (0.98-1.08)	1.01 (0.99-1.04)	1.01 (0.98-1.03)
Substitution of leisure time	1.05 (0.99-1.11)	1.04 (0.98-1.10)	-	1.08 (1.01-1.15) *	1.06 (1.01-1.12) *	1.05 (1.01-1.11) *
Substitution of transportation	0.97 (0.92-1.03)	0.96 (0.91-1.01)	0.92 (0.86-0.99) *	-	0.98 (0.94-1.02)	0.97 (0.93-1.01)
Substitution of sleep	0.99 (0.96-1.02)	0.98 (0.96-1.00)	0.94 (0.89-0.99) *	1.01 (0.97-1.05)	-	0.99 (0.98-1.00)
Substitution of sedentary behavior	1.00 (0.97-1.02)	0.98 (0.96-1.01)	0.94 (0.89-0.99) *	1.02 (0.98-1.06)	1.00 (1.00-1.01)	-
20 minutes						
Substitution of work	-	0.97 (0.91-1.04)	0.89 (0.80-1.00)	1.05 (0.94-1.16)	1.01 (0.96-1.07)	1.00 (0.94-1.05)
Substitution of the home domain	1.02 (0.95-1.09)	-	0.92 (0.81-1.03)	1.07 (0.97-1.18)	1.03 (0.99-1.08)	1.02 (0.97-1.07)
Substitution of leisure time	1.11 (0.99-1.25)	1.08 (0.96-1.22)	-	1.16 (1.01-1.33) *	1.12 (1.01-1.25) *	1.11 (1.01-1.23) *
Substitution of transportation	0.95 (0.85-1.06)	0.93 (0.84-1.02)	0.85 (0.74-0.98) *	-	0.96 (0.89-1.04)	0.95 (0.88-1.03)
Substitution of sleep	0.98 (0.93-1.04)	0.96 (0.92-1.00)	0.88 (0.79-0.98) *	1.03 (0.95-1.12)	-	0.98 (0.97-1.00)
Substitution of sedentary behavior	1.00 (0.94-1.05)	0.97 (0.93-1.02)	0.89 (0.80-0.99) *	1.05 (0.97-1.13)	1.01 (0.99-1.02)	-
30 minutes						
Substitution of work	-	0.96 (0.87-1.07)	0.85 (0.71-1.01)	1.07 (0.91-1.26)	1.02 (0.94-1.10)	1.00 (0.92-1.08)
Substitution of the home domain	1.03 (0.93-1.14)	-	0.88 (0.73-1.05)	1.11 (0.96-1.28)	1.05 (0.98-1.13)	1.03 (0.96-1.10)
Substitution of leisure time	1.17 (0.98-1.39)	1.13 (0.94-1.35)	-	1.26 (1.02-1.54) *	1.19 (1.02-1.40) *	1.17 (1.01-1.37) *
Substitution of transportation	0.93 (0.79-1.09)	0.89 (0.77-1.04)	0.79 (0.64-0.97) *	-	0.95 (0.84-1.07)	0.93 (0.82-1.04)
Substitution of sleep	0.97 (0.90-1.06)	0.94 (0.88-1.01)	0.83 (0.71-0.97) *	1.05 (0.93-1.18)	-	0.97 (0.95-1.00)
Substitution of sedentary behavior	1.00 (0.92-1.08)	0.96 (0.89-1.03)	0.85 (0.72-0.99) *	1.07 (0.95-1.21)	1.02 (0.99-1.04)	-
40 minutes						
Substitution of work	-	0.95 (0.83-1.09)	0.80 (0.64-1.01)	1.10 (0.89-1.36)	1.02 (0.92-1.14)	1.00 (0.89-1.11)
Substitution of the home domain	1.04 (0.91-1.20)	-	0.84 (0.66-1.07)	1.15 (0.95-1.40)	1.07 (0.98-1.17)	1.04 (0.95-1.14)
Substitution of leisure time	1.23 (0.98-1.56)	1.18 (0.93-1.50)	-	1.36 (1.03-1.79) *	1.27 (1.02-1.57) *	1.23 (1.01-1.53) *
Substitution of transportation	0.90 (0.73-1.12)	0.86 (0.71-1.05)	0.73 (0.55-0.96) *	-	0.93 (0.79-1.09)	0.90 (0.77-1.06)
Substitution of sleep	0.97 (0.87-1.08)	0.92 (0.84-1.01)	0.78 (0.63-0.97) *	1.07 (0.91-1.25)	-	0.97 (0.94-1.00)
Substitution of sedentary behavior	1.00 (0.89-1.11)	0.95 (0.87-1.04)	0.80 (0.65-0.99) *	1.10 (0.94-1.29)	1.02 (0.99-1.06)	-
50 minutes						
Substitution of work	-	0.94 (0.79-1.11)	0.76 (0.57-1.02)	1.12 (0.86-1.47)	1.03 (0.90-1.18)	1.00 (0.87-1.14)
Substitution of the home domain	1.05 (0.89-1.25)	-	0.81 (0.60-1.09)	1.19 (0.93-1.52)	1.09 (0.98-1.22)	1.05 (0.94-1.18)
Substitution of leisure timne	1.30 (0.97-1.74)	1.23 (0.91-1.66)	-	1.47 (1.04-2.07) *	1.35 (1.03-1.76) *	1.30 (1.00-1.70)
Substitution of transportation	0.88 (0.67-1.15)	0.83 (0.65-1.06)	0.67 (0.48-0.95) *	-	0.91 (0.75-1.11)	0.88 (0.72-1.08)
Substitution of sleep	0.96 (0.84-1.10)	0.91 (0.81-1.02)	0.73 (0.56-0.96) *	1.09 (0.89-1.32)	-	0.96 (0.93-1.00)
Substitution of sedentary behavior	1.00 (0.87-1.14)	0.94 (0.84-1.05)	0.76 (0.58-0.99) *	1.12 (0.92-1.37)	1.03 (0.99-1.07)	-
60 minutes						
Substitution of work	-	0.93 (0.76-1.14)	0.72 (0.51-1.02)	1.15 (0.84-1.59)	1.04 (0.88-1.23)	1.00 (0.85-1.17)
Substitution of the home domain	1.07 (0.87-1.31)	-	0.77 (0.54-1.11)	1.23 (0.92-1.65)	1.11 (0.97-1.28)	1.07 (0.93-1.22)
Substitution of leisure time	1.37 (0.97-1.95)	1.28 (0.89-1.84)	-	1.59 (1.05-2.40) *	1.43 (1.04-1.98) *	1.37 (1.01-1.89) *
Substitution of transportation	0.86 (0.62-1.19)	0.80 (0.60-1.08)	0.62 (0.41-0.94) *	-	0.90 (0.71-1.14)	0.86 (0.68-1.09)
Substitution of sleep	0.95 (0.81-1.13)	0.89 (0.78-1.02)	0.69 (0.50-0.95) *	1.10 (0.87-1.40)	-	0.95 (0.91-1.00)
Substitution of sedentary behavior	1.00 (0.85-1.17)	0.93 (0.81-1.06)	0.72 (0.52-0.99) *	1.15 (0.91-1.46)	1.04 (0.99-1.09)	-

95%CI: 95% confidence interval; PR: prevalence ratio.

Note: adjusted for sex, age range, hospitalization, family arrangement, depressive symptoms, and frailty.

* p < 0.05.

## Discussion

This study showed that replacing 10, 20, 30, 40, 50, and 60 minutes/day of sleep time and sedentary behavior with moderate-to-vigorous physical activity did not correlate with cognitive decline. However, when an equivalent amount of physical activity replaced sedentary behavior during leisure time, it was a protective factor against cognitive decline in older adults. Although replacing sedentary behavior with other physical activity domains was not a risk factor for cognitive decline, the reallocation of sleep, sedentary behavior, and transport time to physical activity during leisure time was shown to be protective against cognitive decline. However, replacing leisure time by sedentary behavior, sleep, and transport was found to be a risk factor for cognitive decline. The observed protection may be attributed not only to physical activity itself, but also to the context and nature of the activities carried out during leisure time.

Notably, in this study, the substitution of sedentary behavior with moderate-to-vigorous physical activity did not show statistically significant values as a protective factor against cognitive decline. This may be explained by the difficulty of older adults to reach the recommended levels of moderate-to-vigorous physical activity per week ^9^ ^,^ ^34^. This issue leads us to believe that lower levels of physical activity may somehow protect cognitive abilities. A study conducted in Taiwan showed that higher levels of light-intensity physical activity, independent of moderate-to-vigorous physical activity, were associated with a lower rate of cognitive decline ^35^. In contrast, a survey including 1,927 healthy men and women aged 45 to 70 years in the Netherlands showed that the intensity of physical activity, not the total time spent in physical activity, was associated with a reduced risk of cognitive decline ^36^.

The practice of leisure-time physical activity, even at low intensity, can provide physiological benefits, contributing to the preservation of brain function over time ^37^.

The substitution of time spent in sedentary behavior with leisure-time physical activity in the prevention of suggestive cognitive decline in older adults has important physiological implications. The transition from sedentary behavior to leisure-time physical activity is associated with an increase in brain activity, stimulating regions associated with cognition, such as the hippocampus and prefrontal cortex ^38^. Furthermore, regular engagement in leisure-time physical activity promotes improvements in blood circulation, increasing blood flow to the brain ^39^. This increased blood supply facilitates the delivery of oxygen and essential nutrients, contributing to brain health and function ^37^.

In addition, regular engagement in leisure-time physical activity is associated with a reduction in oxidative stress and inflammation, processes that, when excessive, can contribute to suggestive cognitive decline ^40^. Anti-inflammatory and antioxidant mechanisms triggered by physical activity help protect brain cells from damage and degeneration ^41^.

In addition to the benefits of physical activity, both sleep duration and sleep quality are important for organic recovery, with 7-8 hours of sleep per night recommended for older adults ^42^. Sleep quality is directly associated with cognitive performance, influencing functions such as memory, concentration, and decision-making ^16^. However, a systematic review of studies with populations aged ≥ 70 years found that an increase in sleep duration (more than 9 hours per night) was associated with a higher prevalence of cognitive decline ^43^. From this perspective, replacing some sleep time with physical activity during leisure time may be important for preserving cognitive function in older adults.

A systematic review reported an association between increased sedentary behavior and poorer cognition ^15^. Furthermore, a likely mechanism by which sedentary behavior is associated with cognitive decline has been suggested. Recent data suggest that prolonged sedentary behavior impairs glucose and lipid metabolism, which are recognized risk factors for cognitive impairment and all-cause mortality ^44^ ^,^ ^45^. This suggests that sedentary behavior slows down metabolism and contributes to small vessel damage in the brain, which is the underlying mechanism of cognitive decline.

Therefore, active transportation is an interesting alternative to the use of glucose. Furthermore, epidemiological studies have shown that active commuting effectively controls the obesity epidemic and improves the cardiovascular and mental health of the population ^46^ ^,^ ^47^. However, a population-based study conducted in the city of Campinas (Brazil), which analyzed the prevalence of active aging based on the four domains of physical activity according to the IPAQ, found that the prevalence of the transport domain was low (10.9%) compared with the leisure domain (25.3%) ^48^. However, it is noteworthy that unfavorable conditions, such as dangerous streets and sidewalks, can contribute to less activity in the transport domain ^49^. Thus, increasing physical activity during leisure time is of great value, as the major challenge is to get people to reach the recommended levels of physical activity during the week.

Some studies have shown that leisure-time physical activity has a positive effect on the health of older adults. Wang et al. ^50^ found that different leisure activities confer a specific protective factor on different cognitive domains. A high level of physical activity is associated with reduced episodic memory and language. A systematic review showed that leisure-time physical activity in physically active older Chinese adults, particularly those with long-term involvement, is likely associated with a lower risk of cognitive impairment over the years ^51^.

Thus, different types of physical activity, such as stretching ^52^, aerobic exercise ^53^, and resistance training ^54^, have been associated with a lower risk of cognitive decline. Considering the beneficial effect of leisure-time physical activity observed in this study, replacing sedentary behavior with leisure-time physical activity appears to be an effective and feasible method to reduce, prevent, and protect against cognitive decline risk. Therefore, while increased leisure-time physical activity has important implications for cognitive function outcomes, it is certainly possible that the activity for which it is substituted, such as sedentary behavior, sleep, and transport, can influence the magnitude of these effects.

Despite providing relevant findings, this study had some limitations. Although the questionnaires have a good correlation with direct measures, such as the accelerometer ^24^, they are subjective methods. They may lead to overestimation or underestimation of the level of physical activity, as older adults may not accurately recall the hours spent in different behaviors. This is a limitation of this study, which may partly explain why there was no association between the substitution of sedentary behavior with moderate-to-vigorous physical activity and cognitive decline, affecting the quality of the temporal adjustment of the isotemporal substitution method. However, this limitation was mitigated by interviewer training. Furthermore, the isotemporal substitution model is a mathematical method that substitutes one behavior for another and does not represent the actual substitution of behaviors. It is important to note that the results should be interpreted with caution.

Nevertheless, few studies have reported the effects of using an isotemporal substitution model, especially on cognitive decline, which makes our findings crucial despite the limitations of the study. In addition, it can be concluded that both the substitution of physical activity domains and the time spent in specific domains are critical factors, as physical activity alone cannot intervene on cognition. It is also noteworthy that cross-sectional epidemiological studies are important for the development of public health policies.

The findings of this study provide valuable insights for both healthcare professionals and public policy makers in promoting cognitive health in older adults. Replacing prolonged sedentary behavior and sleep with leisure-time physical activity was found to be a protective factor against cognitive decline. Therefore, implementing programs that encourage regular participation in leisure-time physical activity can be an effective intervention. In addition, considering the physiological effects associated with leisure-time physical activity (even at lower intensity), such as brain stimulation and improved blood circulation, can guide specific strategies targeted at the older adult population. It is recommended to promote policies and practices that facilitate the adoption of an active lifestyle during leisure time, thereby contributing to the cognitive health and overall well-being of the older adult population.

## Conclusion

This study suggests that the substitution of sedentary behavior, the transport domain, and sleep with an equivalent amount of leisure-time physical activity is a protective factor against cognitive decline in older adults. In turn, inverse substitution was identified as a risk factor for cognitive decline in older adults. In conclusion, no effects of moderate-to-vigorous physical activity substitution, sedentary behavior, or sleep were observed on cognitive decline.
